# Immunomodulatory Peptides Derived from *Tylorrhynchus heterochaetus*: Identification, In Vitro Activity, and Molecular Docking Analyses

**DOI:** 10.3390/foods15020363

**Published:** 2026-01-20

**Authors:** Huiying Zhu, Zhilu Zeng, Yanping Deng, Jia Mao, Lisha Hao, Ziwei Liu, Yanglin Hua, Ping He

**Affiliations:** 1 College of Food Science and Engineering, Guangdong Ocean University, Yangjiang 529500, China; 2 School of Food Science and Technology, Jiangnan University, Wuxi 214122, China

**Keywords:** *Tylorrhynchus heterochaetus*, peptide, immunomodulatory, RAW 264.7, molecular docking

## Abstract

*Tylorrhynchus heterochaetus* is an aquatic food with both edible and medicinal value in China. With a protein-rich body wall, it has strong potential for producing bioactive peptides. To explore its potential as a source of immunomodulatory peptides, in this study, flavor enzymes were selected as the optimal hydrolases, and the hydrolyzed products were subjected to ultrafiltration fractionation. The <3000 Da portion exhibited the most effective immune-stimulating activity in RAW 264.7 macrophages, enhancing phagocytosis and promoting the secretion of tumor necrosis factor-alpha (TNF-*α*), interleukin-6 (IL-6) and nitric oxide (NO) in a concentration dependent manner. Peptide omics analysis, combined with the activity and safety screened by bioinformatics, identified 43 candidate peptides. Molecular docking predicts that three novel peptides, LPWDPL, DDFVFLR and LPVGPLFN, exhibit strong binding affinity with toll-like receptor 4/myeloid differentiation factor-2 (TLR4/MD-2) receptors through hydrogen bonding and hydrophobic/π stacking interactions. Synthetic verification confirmed that these peptides were not only non-toxic to cells at concentrations ranging from 62.5 to 1000 µg/mL, but also effective in activating macrophages and stimulating the release of immune mediators. This study successfully identified the specific immunomodulatory peptides of the *Tylorrhynchus heterochaetus*, supporting its high-value utilization as a natural source of raw materials for immunomodulatory functional foods.

## 1. Introduction

*Tylorrhynchus heterochaetus (T. heterochaetus)*, commonly known as “Hechong” in China, belongs to the *phylum Annelida*, *class Polychaeta*, *order Nereididae*, and *family Nereida* [1]. This species is primarily distributed in the mangrove wetlands of the Pearl River Delta, China, where freshwater and saltwater converge [2]. It is a benthic invertebrate aquatic animal. Similar to traditional aquatic resources such as oysters, *T. heterochaetus* is valued not only as food but also as a traditional remedy used to improve physical weakness, relieve fatigue, or spleen and stomach function in China. Modern pharmacological research has further demonstrated its diverse biological activities. For instance, its water extract can prolong loaded swimming time in mice, reduce blood lactic acid levels, and increase liver glycogen by activating the nuclear factor erythroid 2-related factor 2/antioxidant response element (Nrf2/ARE) signaling pathway, all of which demonstrate its beneficial anti-fatigue effect. In addition, its enzymatic hydrolysate exhibits antioxidant activity [3,4]. *T. heterochaetus* is rich in nutrients, as it contains high-quality proteins, various amino acids, and essential trace elements. With a protein content of approximately 65–78% (dry weight), it is an ideal raw material for producing bioactive peptides. However, current research on *T. heterochaetus* has mainly focused on mitochondrial genome analysis and hemoglobin structure. In-depth exploration of its protein resources, particularly bioactive peptides with specific functions, remains scarce [5,6,7,8,9]. Therefore, the systematic discovery of immunomodulatory bioactive peptides from *T. heterochaetus* not only holds significant theoretical value but also offers broad application prospects [10].

The immune system can maintain physiological homeostasis through a dynamic, multi-level defense network [11]. It performs essential functions, including pathogen clearance, surveillance of abnormal cells, and regulation of immune balance. However, with increasing environmental pollution, stress, and an aging population, the incidence of immune-related diseases continues to rise. It is estimated that autoimmune diseases affect approximately 3% of the global population, and in some regions, the figure is even higher. Such diseases include rheumatoid arthritis, systemic lupus erythematosus, multiple sclerosis, etc. [12]. Chronic inflammatory conditions and immune dysfunctions further contribute to the burden of non-communicable diseases, posing a global public health challenge [13]. Although immune-enhancing drugs (e.g., recombinant interleukin-2 and levamisole) are available, their long-term health risks and adverse reactions, including the risk of triggering autoimmune reactions, cytokine release syndrome, and organ toxicity, remain a concern. Therefore, developing safe, efficient, and naturally derived immune-regulating active ingredients is urgently needed.

In this context, immunomodulatory peptides—short protein fragments typically comprising 2–20 amino acid residues that can regulate immune cell activity, influence cytokine production, and modulate signaling pathways—have emerged as promising candidates. These peptides can be derived from various natural sources and are broadly classified based on their origin. Marine-derived peptides, for instance, have shown remarkable immunomodulatory potential. Examples include peptides from fish sources, such as those from tuna hydrolysates, which have been reported to enhance macrophage phagocytosis and stimulate nitric oxide (NO) production [14]. Similarly, peptides derived from shrimp or shellfish can influence the secretion of both pro- and anti-inflammatory cytokines [15,16].

Immune-regulating active peptides derived from natural products offer unique advantages in functional foods and immune adjuvant therapies, owing to their high specificity, potent bioactivity, and favorable safety profiles. In this study, *T. heterochaetus* was used as the raw material. Enzymatic hydrolysates exhibiting immunomodulatory activity were prepared through enzymatic hydrolysis, and their peptide sequences were identified and characterized. Virtual screening was then conducted through computer-based simulations and molecular docking. Peptides showing strong predicted activity were synthesized using solid-phase peptide synthesis, and their immunological activity was verified in RAW 264.7 macrophages. Finally, molecular docking was employed to elucidate the interaction mechanisms between the identified immunomodulatory peptides and the toll-like receptor 4/myeloid differentiation factor-2 (TLR4/MD-2) receptor in macrophages. Together, this study provides a theoretical foundation and scientific evidence for the high-value utilization of *T. heterochaetus*.

## 2. Materials and Methods

### 2.1. Materials and Reagents

*T. heterochaetus* (fresh) was purchased from Hechong Science and Technology Park, Yangjiang, China. Alkaline protease (200 U/mg), neutral protease (100 U/mg), and flavorzyme (150,000 U/g) were obtained from Shanghai Macklin Biochemical Co., Ltd., (Shanghai, China). Papain (800 U/mg), trypsin (250 U/mg), and bromelain (1200 U/mg) were obtained from Shanghai Yuanye Bio-Technology Co., Ltd., (Shanghai, China). The murine macrophage RAW 264.7 cell line was provided by the Cell Bank of the Chinese Academy of Sciences (Shanghai, China). High-glucose Dulbecco’s modified eagle medium (DMEM), sterile phosphate-buffered saline (PBS), and 0.25% trypsin were purchased from Gibco (Carlsbad, CA, USA). Penicillin/streptomycin solution, fetal bovine serum (FBS), and the Cell counting kit-8 (CCK-8) were purchased from Dalian Meilun Biotechnology Co., Ltd., (Dalian, China).

### 2.2. Basic Nutrient Component Determination in T. heterochaetus, Enzyme Screening and Sample Preparation

The following components in *T. heterochaetus*, such as protein, ash, fat, and moisture, were analyzed according to the method of He et al. [17]. and the AOAC Official Methods. The experimental procedure was adapted from Xia et al. [18] with minor modifications. Fresh *T. heterochaetus* was mixed evenly with distilled water in a 1:1 (*w*/*v*) ratio in a tissue homogenizer (Shuibei Youlian Instrument Factory, Jintan District, Changzhou, China). To screen the most effective hydrolytic enzyme, six proteases, including alkaline, neutral protease, bromelain, flavorzyme, papain, and trypsin, were selected as candidates. Enzymatic hydrolysis reactions were carried out under the optimal temperature and pH specific to each enzyme. The optimal reaction conditions of each protease are shown in Table 1.

After hydrolysis, the samples were heated in a boiling water bath (HWS-26 constant temperature water bath, Shanghai Hengke Science Instrument Co., Ltd., Shanghai, China) for 15 min to inactivate the enzymes. After cooling, the mixtures were centrifuged at 6813× *g* for 20 min at 4 °C using a high-speed refrigerated centrifuge (MA600R, Shanghai Fengbo Biotechnology Co., Ltd., Shanghai, China). The supernatant was filtered and collected as the enzymatic hydrolysate of *T. heterochaetus*. The degree of hydrolysis (DH) was determined, the effects of each hydrolysate on the secretion of cytokine tumor necrosis factor-alpha (TNF-*α*) were examined, and the optimal protease was identified. The enzymatic hydrolysis products of the optimal protease (THPs) were subjected to ultrafiltration using a 3000 Da ultrafiltration membrane, and the permeate (THPs-I, <3000 Da) and retention (THPs-II, >3000 Da) were collected for further analysis.

### 2.3. Determination of DH

The DH was calculated by the following equation [19]:

(1) $$DH\left(\%\right)=\frac{N_{2}-N_{1}}{N_{0}-N_{1}}\times 100\%$$

where N_0_ is the total nitrogen content in *T. heterochaetus* flesh; N_1_ is the ammoniacal nitrogen content in the flesh; N_2_ is the ammoniacal nitrogen content of the enzymatic hydrolysate (g/g).

### 2.4. Amino Acid Composition Analysis

Amino acid composition was determined following the method described in Fu et al. [20] with slight modifications. Freeze-dried sample (0.5 g) was mixed with 10 mL of 6 mol/L HCl and hydrolyzed at 110 °C for 24 h. The hydrolyzed was filtered and diluted with water. An aliquot of 1.0 mL was dried under nitrogen and reconstituted in 1.0 mL of 0.02 mol/L HCl. The solution was filtered through a 0.22 μm filter membrane and analyzed using an automatic amino acid analyzer (LA8080, Hitachi, Tokyo, Japan).

### 2.5. Determination of Molecular Weight Distribution

The molecular weight (M_w)_ distribution was determined according to the method described by Zhao et al. [21] with minor modifications. Analysis was performed by high-performance gel permeation chromatography using an Agilent 1260 system (Agilent Technologies, Santa Clara, CA, USA) equipped with a TSK gel filtration column (30 cm × 7.8 mm inner diameter). The mobile phase consisted of acetonitrile:water:trifluoroacetic acid (20:80:0.1) at a flow rate of 0.5 mL/min. The column temperature was set at 25 °C, and the sample volume was 20 μL. UV detection was performed at 220 nm. Standards were prepared using insulin (M_w_: 5778 Da), bacitracin (M_w_: 1422 Da), Gly-Gly-Tyr-Arg (M_w_: 451 Da), and Gly-Gly-Gly (M_w_: 189 Da). A calibration curve was constructed by plotting retention time of the standards against their M_w_ and was used to estimate the M_w_ distribution of samples.

### 2.6. Cell Culture

Cells were cultured in DMEM medium containing 10% FBS and 1% (*v*/*v*) penicillin-streptomycin at 37 °C in an incubator humidified with 5% CO_2_. Cells between passages 15 and 25 were used for all experiments. All experiments were performed using cells in the log phase.

### 2.7. Evaluation of Cytotoxicity in Murine RAW 264.7 Macrophages

The cytotoxicity of the samples toward RAW 264.7 macrophages was evaluated using the CCK-8 assay, following Jiang et al. [22] with slight modifications. Cells in log phase were cultured at a density of 1 × 10^5^ cells/well in 96-well plates. The medium was then replaced with fresh medium containing the samples at different concentrations, and the cells were further cultured for 24 h. Culture medium without samples was used as the negative control. After removing the supernatant, 10% CCK-8-containing reagent was added, and the cells were incubated for 1 h. The absorbance was measured at 450 nm using the iD3 multi-functional microplate reader (MegaGene Molecular Company, Sunnyvale, CA, USA). Cell viability was calculated as follows:

(2) $$Cell\;viability\;(\%)=\frac{A_{S}}{A_{C}}\times 100$$

where A_S_ is the absorbance of the sample group, and A_C_ is the absorbance of the negative control group.

### 2.8. Determination of Phagocytic Activity

Phagocytic activity was assessed following the method of Wang Zhen et al. [23] with slight modifications. Cells were cultured and treated with samples as described in Section 2.7. After a 24-h treatment period, the phagocytic capacity of the cells was determined using a commercial Neutral Red phagocytosis kit.

### 2.9. Determination of TNF-α,Interleukin-6 Cytokine Expression Level, and NO Production

The levels of secreted immune mediators were assessed as follows [24]. RAW 264.7 macrophages were cultured and treated with samples according to the procedure outlined in Section 2.7. The concentration of TNF-α in the culture supernatant was determined using a mouse TNF-α ELISA kit. The concentration of interleukin-6 (IL-6) was also measured using a mouse IL-6 ELISA kit. The production of NO was determined using a NO detection kit.

### 2.10. Peptide Sequence Identification Using LC-MS/MS

Peptide samples were first subjected to desalting using ZipTip C18. Samples were dissolved in 0.1% trifluoroacetic acid (TFA). The ZipTip C18 was rinsed 10 times with 50 μL of a solution consisting of 60% acetonitrile (ACN) and 0.1% TFA, followed by washing 10 times with 10 μL of 0.1% TFA. The sample solution was aspirated and dispensed through the ZipTip 20 times, after which the liquid was discarded. The ZipTip was then washed five additional times with 10 μL of 0.1% TFA. Finally, the peptides were eluted with 10 μL of a solution containing 60% ACN and 0.1% TFA into a new Eppendorf (EP) tube and dried under vacuum.

The desalted peptides were analyzed by liquid chromatography-tandem mass spectrometry (LC-MS/MS; Thermo, Waltham, MA, USA). Chromatographic separation was performed on a 75 μm × 150 mm PepMap RSLC C18 column (2 μm, 100 Å). Mobile phase A consisted of 0.1% formic acid in water, and mobile phase B consisted of 0.1% formic acid in acetonitrile. The LC gradient was as follows: 0–50 min, 95–60% A, 5–40% B; 50–65 min, 60–5% A, 40–95% B; and 65–70 min, 5–95% A, 95–5% B. The flow rate was 250 nL/min. Samples were separated by a capillary high-performance liquid chromatography (HPLC) and analyzed using a Thermo Scientific Q Exactive mass spectrometer. Peptide identification was carried out using PEAKS13.1 software.

### 2.11. Computer Simulation Screening

The biological activities of the identified peptide sequences were predicted using the PeptideRanker database (http://distilldeep.ucd.ie/PeptideRanker/, accessed on 15 December 2025). Peptides with predicted activity scores > 0.5 were considered potential bioactive peptides [25]. Toxicity and allergenicity of the identified peptides were assessed using the ToxinPred database (https://webs.iiitd.edu.in/raghava/toxinpred/index.html, accessed on 15 December 2025) and the AllergenFP v.1.0 database (ddg—pharmfac.net/AllergenFP/, accessed on 15 December 2025). Finally, use ADMETlab 3.0 (https://admetlab3.scbdd.com/server/screening, accessed on 15 December 2025) for forecasting Caco-2 cell permeability [26].

### 2.12. Molecular Docking

Molecular docking was conducted following the method of Jiang et al. [22] with minor modifications using AutoDock Vina 1.5.7. The binding affinities between the peptides and the TLR4/MD-2 complex were estimated. The crystal structure of the TLR4/MD-2 (PDB ID: 5IJD) was retrieved from the Protein Data Bank (https://www.rcsb.org/, accessed on 15 December 2025). During docking, the receptor structure was kept rigid, while the peptide structures were treated as flexible. Candidate peptides were evaluated and screened based on docking binding energy and relevant physicochemical properties.

### 2.13. Synthesis and Activity Validation of Predicted Bioactive Peptides

The peptides LPWDPL, DDFVFLR, and LPVGPLFN were synthesized using the solid-phase synthesis method. The peptide purity was >95%. Their immunomodulatory activities were evaluated using the assays described in Section 2.7, Section 2.8 and Section 2.9.

### 2.14. Statistical Analysis

All experiments were repeated 3 times. Data were organized using Excel 2019. Graphs were generated using Origin 2022. Statistical analyses were performed using SPSS Statistics 19. Results are expressed as mean ± standard deviation (SD). Student’s *t*-test was used to compare the data between the two groups, and *p* < 0.05 was considered statistically significant.

## 3. Results and Discussion

### 3.1. Selection of Proteases

**Figure 1:**
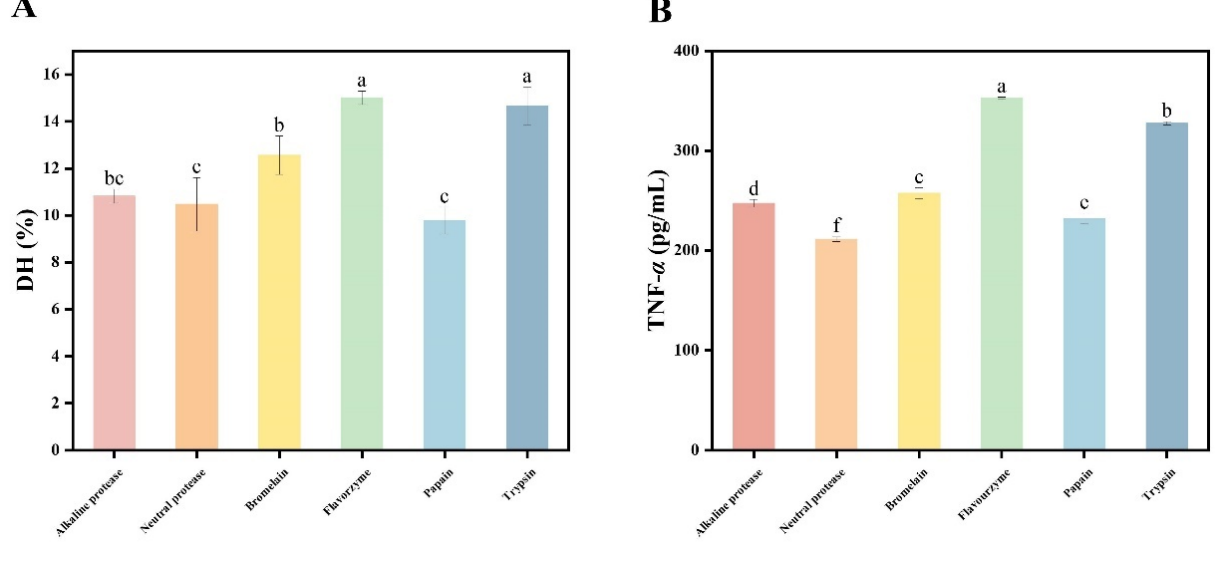
Effects of *Tylorrhynchus heterochaetus* hydrolysate derived from different proteases on the DH and cytokine TNF-*α*. (**A**) DH. (**B**) Cytokine TNF-*α*. Different lowercase letters above bars indicate significant differences (*p* < 0.05).

As shown in Table 2, the protein content of the fresh *T. heterochaetus* used in this experiment is 12.54 ± 0.50%, the fat content is 3.07 ± 0.05%, the ash content is 0.69 ± 0.03%, and the moisture content is 81.82 ± 0.05%. This is an ideal raw material for preparing functional active peptides. DH is a key indicator for evaluating the catalytic efficiency and reaction progress of proteases [27]. The homogenate of *T. heterochaetus* was hydrolyzed with six proteases: alkaline protease, trypsin, papain, bromelain, flavorzyme, and neutral protease. As shown in Figure 1A, the DH values differed significantly among the enzymatic hydrolysates (*p* < 0.05). Flavorzyme and trypsin had the highest DH values, 15.01% and 14.65%, respectively. This shows that they have strong proteolytic capabilities. The DH of bromelain was at a moderate level. The hydrolytic efficiency of alkaline protease, neutral protease, and papain was relatively low. Among all, papain had the lowest DH value, suggesting that it has limited ability to cleave peptide bonds under experimental conditions.

The effect of the enzymatic hydrolysate on the cytokine TNF-*α* is shown in Figure 1B. The flavorzyme hydrolysate significantly promoted the secretion of TNF-*α*. The highest TNF-*α* level was approximately 352 pg/mL, which was significantly different from that of the other groups (*p* < 0.05). The second-highest TNF-*α* level was from trypsin; however, the value was significantly lower compared to flavourzyme but notably higher compared to other groups. Bromelain and alkaline protease resulted in moderate TNF-*α* levels, and the values for these two groups were statistically significantly different. Among all tested proteases, papain and neutral protease resulted in the lowest TNF-*α* levels, and neutral protease was the least effective inducer. Based on both DH values and TNF-*α* levels, flavorzyme was selected as the optimal protease for hydrolyzing *T. heterochaetus* proteins.

### 3.2. Amino Acid Composition

The amino acid composition of different THPs is summarized in Table 3. The predominant amino acids included glutamic acid (8.19%), aspartic acid (5.70%), and lysine (5.13%). Hydrophobic amino acids (Gly, Ala, Val, Ile, Leu, Phe, His and Trp), branched-chain amino acids (Val, Ile and Leu), aromatic amino acids (Tyr, Phe and Trp), basic amino acids (His, Lys and Arg) and acidic amino acids (Asp and Glu) in THPs accounted for 24.70%, 11.39%, 6.85% and 13.89%, respectively. Both the composition and types of amino acids were associated with biological activity [28]. Studies have shown that, as essential amino acids for the human body, branched-chain amino acids (BCAAs) are not only common components of immunomodulatory peptides, but also play a significant role in the catabolism and energy supply of the three major nutrients [29,30,31,32]. Therefore, obtaining adequate BCAAs from food-derived proteins is important for physiological functions. Hydrophobic amino acids have also been reported to be important components of immunomodulatory peptides. Hydrophobic amino acids can enhance the immune activity of peptides by increasing their affinity in cell membranes and improving their recognition efficiency by immune cell receptors. Consequently, peptides containing hydrophobic residues, such as valine and leucine at the terminal end, often exhibit good activity [33,34,35]. THPs is similar to the three novel immunomodulatory peptides prepared from the *Rana spinosa*, which are rich in various hydrophobic amino acids, suggesting its potential to enhance immune activity [13].

### 3.3. Mw Distribution

Bioactive peptides are believed to be short peptides containing 2 to 10 amino acid residues, and their Mw plays a critical role in determining their activity [36]. The Mw distribution of THPs is presented in Table 4. Results showed that 91.38% of the peptides had an Mw below 3000 Da, among which 57.65% had an Mw between 500 Da and 1000 Da. Zhang et al. [37] reported that the Mw of antioxidant peptides in walnut protein hydrolysates was predominantly <1000 Da. Similarly, some scholars demonstrated that the Mw of immunomodulatory peptides from yellowfin tuna was mainly distributed <3000 Da [38].

### 3.4. Immunomodulatory Activity of Enzymatic Hydrolysates

#### 3.4.1. Influence on Cell Viability and Phagocytic Capacity

**Figure 2:**
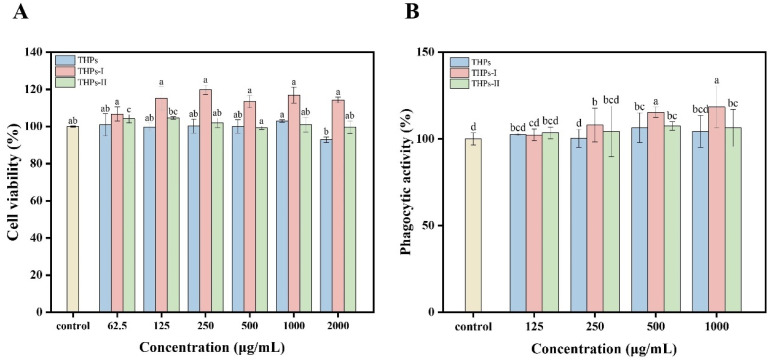
Effects of *T. heterochaetus* hydrolysates on the immunomodulatory activity of RAW 264.7 macrophages: (**A**) cell viability, and (**B**) phagocytic activity. Different letters above bars indicate significant differences (*p* < 0.05).

Before evaluating the immunomodulatory ability of the *T. heterochaetus* hydrolysates, their effects on RAW 264.7 cell viability were first evaluated. As shown in Figure 2A, THPs, THPs-I, and THPs-II at the concentration range of 62.5–2000 μg/mL showed no toxicity to RAW 264.7 cells; rather, they promoted their proliferation. Among all, the constituents of THPs-I show a significant value-added ability. Macrophages in a dormant state exhibit relatively low phagocytic capacity; therefore, an increase in phagocytic activity can be used as an indicator of macrophage activation [39]. As shown in Figure 2B, at a concentration range of 250–1000 μg/mL, THPs-I significantly enhanced the phagocytic activity of RAW 264.7 cells. At 1000 μg/mL, THPs-I increased phagocytic activity to 118.39 ± 3.09%, which was significantly higher than that of the control, THPs, and THPS-II groups (*p* < 0.05). Phagocytosis is the primary defense mechanism through which macrophages eliminate invading pathogens and maintain immune homeostasis [40]. The observed enhancement of phagocytic activity indicates that THPs-I possesses immunomodulatory capacity and can promote the phagocytic activity of macrophages.

#### 3.4.2. Effects on Cytokine Secretion

**Figure 3:**
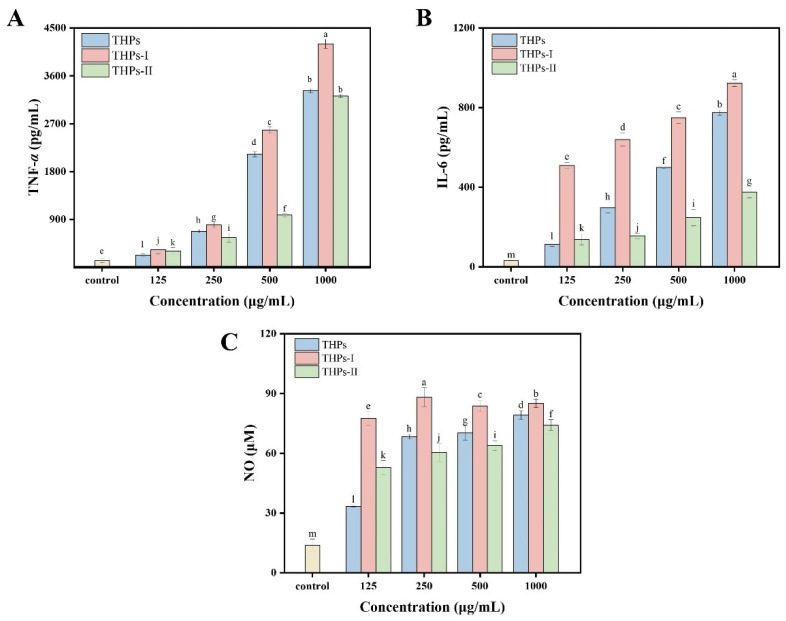
Effects of the *T. heterochaetus* hydrolysates on immune factors in RAW 264.7 cells: (**A**) TNF-*α* content. (**B**) IL-6 content, and (**C**) NO content. Different letters above bars indicate significant differences (*p* < 0.05).

Activated macrophages can participate in immune responses by secreting immune factors and NO, which are crucial for protecting against foreign pathogens [41]. As shown in Figure 3A, at 1000 μg/mL, THPs-I and THPs-II significantly increased the level of TNF-*α* from 1284.41 pg/mL to 4203.25 and 3324.07 pg/mL, respectively. This level is higher than the TNF-*α* level secreted by soybean peptides [40] and the enzymatic hydrolysis products of bullfrogs [13] at the same concentration. As shown in Figure 3B, THPs-I compared to the control group, the secretion of IL-6 was significantly increased at concentrations of 125 to 1000 μg/mL (*p* < 0.05). It is worth noting that at each concentration, the secretion of IL-6 caused by THPs-I was significantly higher compared to the other two samples. This indicates that the low-molecular-weight peptide has the strongest promoting effect on IL-6 secretion in RAW 264.7 cells. Moreover, as the concentration of this peptide increased, the secretion of IL-6 increased until reaching the maximum value at a concentration of 1000 μg/mL. This result suggests that the immune effect is concentration-dependent. Studies have shown that NO can not only directly activate the phagocytic activity of macrophages but also stimulate them to secrete cytokines, thereby synergistically amplifying overall immune activity [42]. As shown in Figure 3C, the NO-promoting effects of all three samples were concentration-dependent. All samples could induce RAW 264.7 to secrete NO, and the higher the concentration, the stronger the NO-promoting ability. At 1000 μg/mL, the activity of the hydrolysate of all three groups was significantly higher than that at the same concentration [22]. Among all samples, THPs-I could most effectively stimulate NO production, especially at a low concentration (125 μg/mL). The NO production reached 77.58 μM, which was significantly higher compared to other groups. This finding suggests that THPs-I contain immune-stimulating substances and thereby could activate macrophages and increase the release of the immune mediator NO.

### 3.5. Peptide Sequence Identification, Computer Simulation Screening, and Molecular Docking

Peptides in THPs-I were identified by LC-MS/MS. Peptides with an acceptable level of confidence (ALC) ≥98% were preferentially screened, and 193 high-confidence peptides were obtained. Immunomodulatory active peptides were screened from these peptides using computer simulation in combination with molecular docking. First, bioactivity was predicted using an online tool, PeptideRanker. Peptides with a score of ≥0.5 are considered to exhibit biological activity. Further safety assessment of peptides was conducted. ToxinPred and AllergenFP v.1.0 were used to evaluate toxicity and sensitization, respectively. Hydrophobicity was calculated based on the Kyte-Doolittle scale. The intestinal absorption potential was predicted using a computer model for Caco-2 cell permeability. Finally, the binding affinity (reflected by the binding energy) between the peptide and the TLR4/MD-2 receptor was determined. The most promising immunomodulatory active peptides were identified based on the above parameters. Among the 193 peptides identified with ALC ≥ 98%, 43 peptides had a PeptideRanker bioactivity score of ≥0.5. The predicted physicochemical properties and binding energies of these peptides are summarized in Table 5. All 43 peptides exhibited high biological activity and ALC. Among all, the peptide with the best performance was LPWDPL (biological activity score: 0.93513, ALC: 98.7%). In addition, all 43 peptides were predicted to be non-toxic and non-allergenic; thus, they may be employed as functional foods or drugs.

As shown in Table 5, the hydrophobicity distribution range of these peptides ranged from −0.40 to 0.23. Peptides with moderate hydrophobicity are generally considered to have better membrane interaction and permeation potential. To further assess absorption characteristics, the Caco-2 cell penetration model was used. The predicted apparent permeability coefficient values of the peptides ranged from −9.167 to −5.609. In general, peptides with higher permeability are more easily absorbed by the intestines and thus are more likely to have a greater impact on the body. Taken together, the comprehensive analysis of hydrophobicity and permeability indicated that LPWDPL and DDFVFLR peptides possessed favorable oral bioavailability; thus, they could be developed into functional ingredients.

TLR4 is widely expressed in innate immune cells, including macrophages, and immunomodulatory peptides can regulate the downstream signaling factors of RAW 264.7 by binding to TLR4 on its cell membrane [43,44,45]. To explore this mechanism, molecular docking was performed between TLR4/MD-2 and the 43 identified peptides. Based on binding affinity rankings, the top three peptides were LPWDPL, DDFVFLR, and LPVGPLFN, with binding energies of −9.1, −9.0, and −9.0 kcal/mol, respectively. All three peptides have binding energies ≤−9 kcal/mol. Because binding free energies below −5 kcal/mol typically indicate spontaneous and favorable ligand-receptor interactions, these results suggest that the three peptides exhibit strong and stable affinity for the TLR4 receptor and may therefore exhibit immunomodulatory activity [46]. A comprehensive assessment of the physicochemical properties and functional characteristics of the three peptides was further carried out. PeptideRanker prediction scores of all peptides were above 0.65, suggesting that they may potentially possess functional activity. Considering their safety, none of the peptides exhibited toxicity or sensitization, and their ALC values were higher than 98.7%, indicative of excellent biocompatibility. For absorption characteristics, the hydrophobicity (GRAVY value) of the three peptides ranged from −0.13 to 0.20. These numbers show that the peptides had moderate hydrophobicity, which is conducive to membrane interaction. Moreover, the predicted Caco-2 permeability values ranged from −7.892 to −6.449, suggesting that they could be absorbed by the intestine. In summary, based on the above data, the peptides LPWDPL, DDFVFLR, and LPVGPLFN were selected for subsequent in vitro immunoregulatory validation.

**Figure 4:**
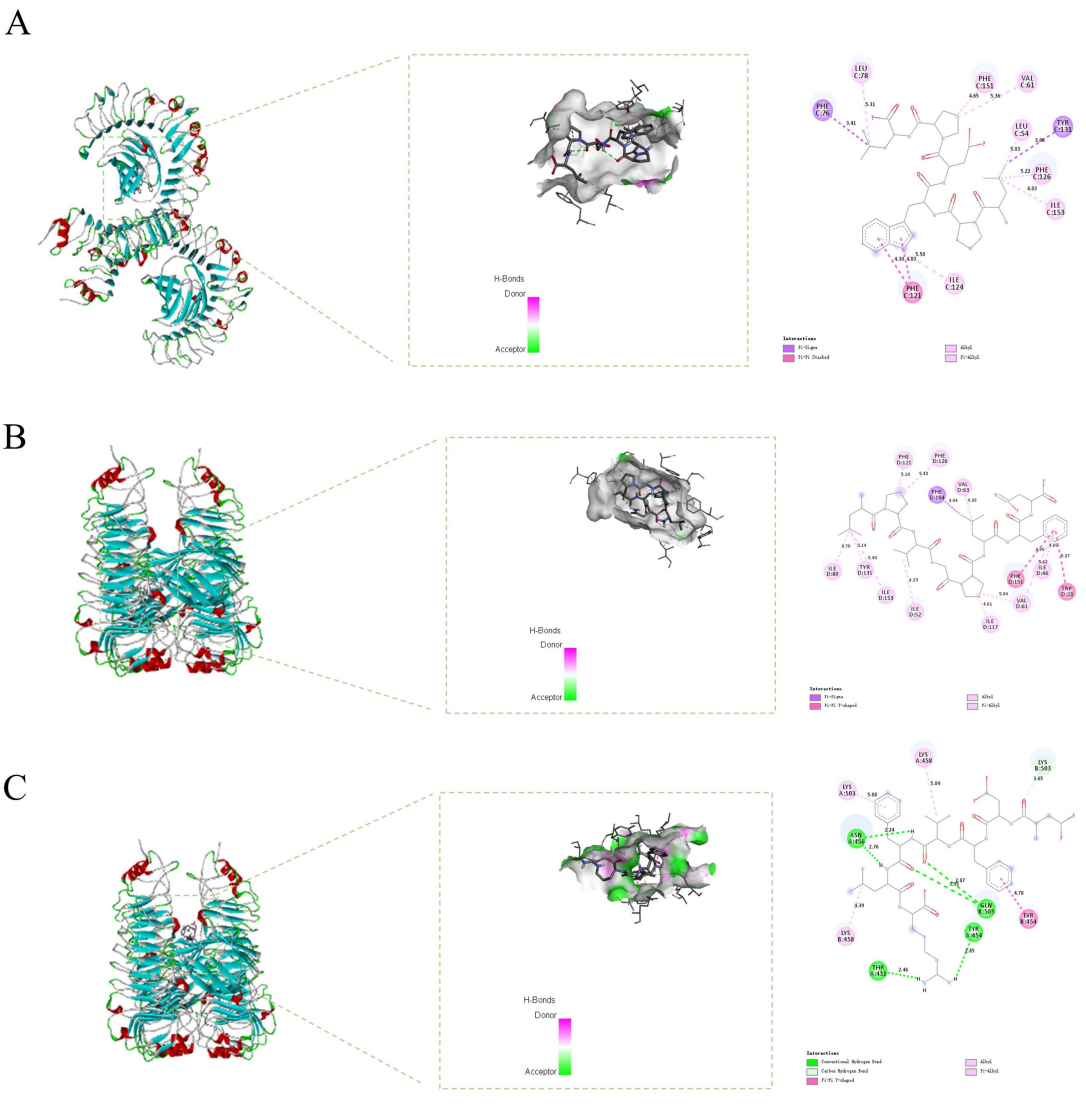
Molecular docking of three peptides with the TLR4/MD-2 complex: (**A**) LPWDPL; (**B**) LPVGPLFN; (**C**) DDFVFLR. Left: the overall structure of the complex. Middle: detailed interactions between the peptide and the receptor. Right: 2D diagram showing the interaction sites between the peptide and the receptor.

Analysis of the interactions between the three peptides and the TLR4/MD-2 complex is shown in Figure 4. The interaction sites between LPWDPL and TLR4/MD-2 are shown in Figure 4A. The aromatic ring of LPWDPL forms π-π stacking interactions with Phe 121 of TLR4/MD-2 with distances of 4.30 and 4.03 Å. Such π-electron interactions between aromatic rings provide strong hydrophobic stabilization and enhance the binding specificity of peptides to receptors. In addition, multiple hydrophobic residues of LPWDPL interact with Leu 78, Phe 151, Val 61, Phe 126, and Ile 153 of TLR4/MD-2, with interaction distances ranging from 3.41 to 5.31 Å. The abundance of hydrophobic interactions suggests that LPWDPL can stably occupy the MD-2 hydrophobic pocket. The binding may change the conformation of the intracellular domain of TLR4, affect the activation degree of immunoregulatory cells, inhibit the activation of the TLR4-mediated NF-κB pathway, and downstream release of pro-immune factors (such as IL-6 and TNF-*α*). This finding provides valuable insights into pathogenesis, offering potential treatment strategies for immune-related diseases. As shown in Figure 4B, the aromatic residues of LPVGPLFN also form π-π stacking interactions with Phe 151 and Trp 23 of MD-2, with distances of 4.95 and 5.27 Å. These interactions can significantly enhance the specificity of the peptide to the receptor and are among the core mechanisms for TLR4 ligand recognition. It also indicates that these interactions are a common pattern of food-derived immune peptides activating the TLR4 pathway. LPVGPLFN also forms hydrophobic interactions with Ile 117, Val 61, Ile 52, Tyr 131, Ile 80, Val 63, Phe 126, and Phe 121 of MD-2. In contrast, DDFVFLR exhibited polar binding characteristics. It forms hydrogen bonds with Asn 456, Thr 431, Tyr 454, and Gln 505 of TLR4, as well as carbon-hydrogen bonds with Lys 503 (Figure 4C). These directional interactions significantly enhance the stability and specificity of the binding [47]. Furthermore, a Pi-Pi T-shaped packing was observed between DDFVFLR and Tyr 454, further reinforcing its spatial positioning within the binding pocket. The results of molecular docking confirmed that it was precisely these residues that directly mediated the interaction with the key sites of the receptor. This provides direct molecular-level evidence for the structure-activity relationship where the composition of specific amino acids determines activity. Overall, the three identified peptides exhibited potential immunomodulatory activity by binding to the TLR4/MD-2 receptor via multiple non-covalent interactions.

### 3.6. Verification of Activities of Synthetic Peptides

#### 3.6.1. Effects of Synthetic Peptides on Proliferation and Phagocytic Activity of RAW 264.7 Macrophages

**Figure 5:**
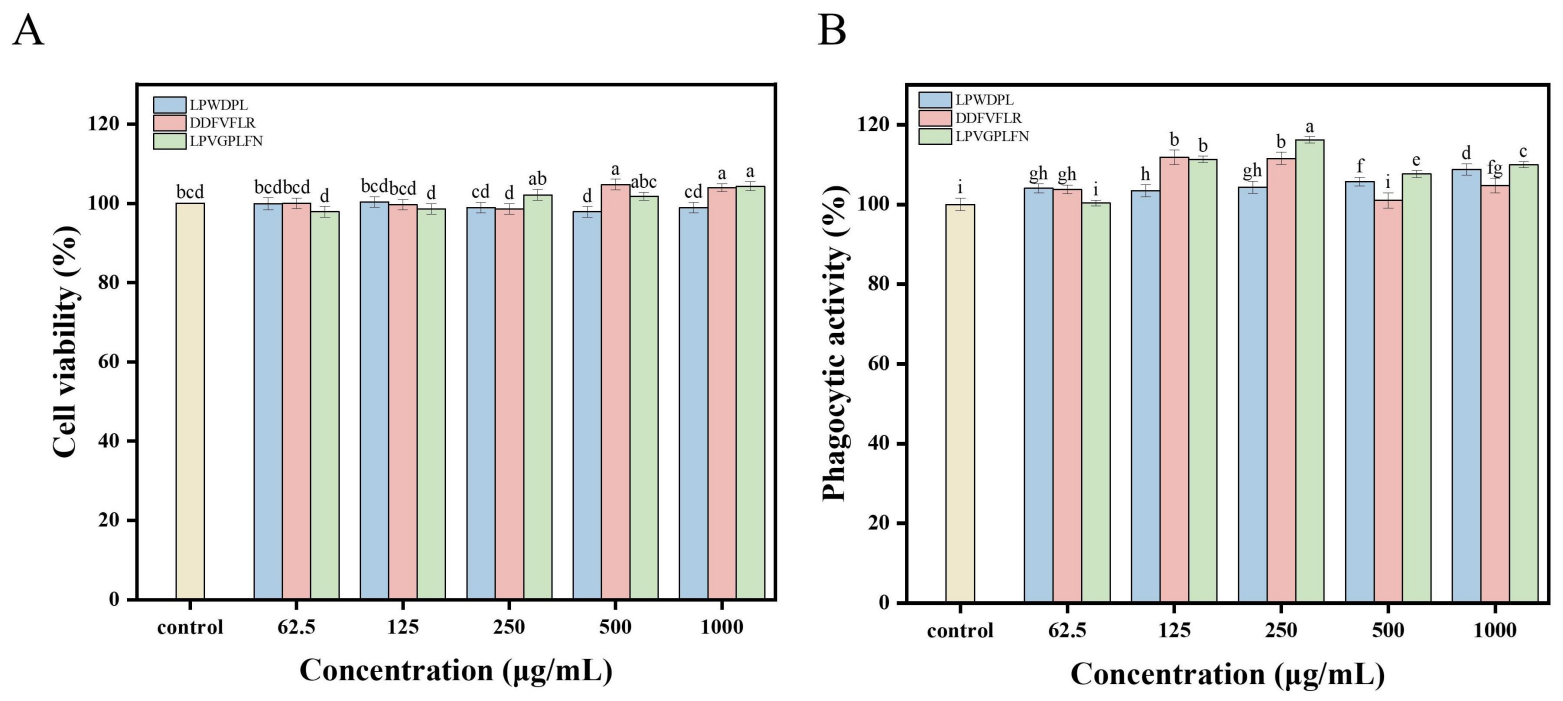
Effect of synthetic peptides on immunological activity of RAW2 64.7 macrophages: (**A**) cell viability, and (**B**) phagocytic activity. Different letters above bars indicate significant differences (*p* < 0.05).

To validate the computational screening results, the top three peptides, including LPWDPL, DDFVFLR, and LPVGPLFN, were synthesized using solid-phase peptide synthesis. Their immunomodulatory effects on cell survival rate and phagocytic activity of RAW 264.7 macrophages were assessed. The cytotoxicity results (Figure 5A) showed that, with peptides at concentrations of 62.5–1000 μg/mL, cell viability in most peptide-treated groups was not significantly different from the control group (*p* > 0.05). This finding indicates that all three peptides were not cytotoxic at the determined concentration range and thus had good biocompatibility. It is worth noting that at concentrations of 500 μg/mL and 1000 μg/mL, the viability of cells in the DPNFDLR-treated group was significantly higher than that of the control group (*p* < 0.05), reaching the highest of 104.74% at 500 μg/mL, which is 1.05 times that of the control group. Likely, this peptide can more effectively promote cell proliferation or have better biocompatibility compared to other peptides. The viability of cells in both the LPWDPL and LPVGPLFN groups was relatively high, further indicating that these cells could tolerate the peptides within the tested concentration range. Considering the phagocytic activity (Figure 5B), LPWDPL and DDFVFLR could significantly enhance phagocytic activity at a relatively low concentration of 62.5 μg/mL, reaching 104.03% and 103.75%, respectively (*p* < 0.05). As the concentration was increased (125 μg/mL and 250 μg/mL), the phagocytic activities of cells in the DPNFDLR and LPVGPLFN groups further increased (*p* < 0.05), and the values were significantly higher than those of the LPWDPL and control groups. At 250 μg/mL, the phagocytic activities of the two peptides reached their peak values at 111.52% and 116.22%, respectively. Even at a high concentration of 1000 μg/mL, all three samples could maintain high phagocytic activity, indicating that they can promote phagocytosis at a wide concentration range.

Collectively, none of the three synthetic peptides showed obvious cytotoxicity within the tested concentration range and could effectively enhance the phagocytic activity of macrophages. These peptides could potentially be utilized as immunomodulatory and functional enhancement agents; however, further in-depth research is required.

#### 3.6.2. Effect of Synthetic Peptides on Cytokine Secretion

**Figure 6:**
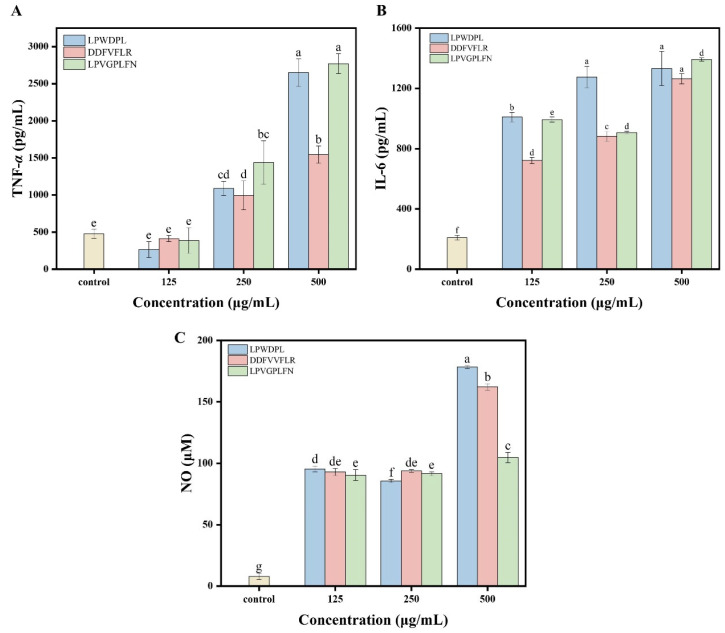
Effects of synthetic peptides on immune mediator factors in RAW 264.7 macrophages: (**A**) TNF-*α* content; (**B**) IL-6 content; and (**C**) NO content. Different letters above bars indicate significant differences (*p* < 0.05).

The influences of three synthetic peptides on the secretion of pro-immune cytokines (TNF-*α* and IL-6) and NO production in RAW 264.7 macrophages were further evaluated. As shown in Figure 6A–C, at concentrations of 250 and 500 μg/mL, all three synthetic peptides could significantly promote the secretion of TNF-*α*. Compared to the untreated control group, the IL-6 secretion levels and NO production of the peptide-treated groups were significantly higher (*p* < 0.05) at all tested concentrations. The release of IL-6 was concentration-dependent. For instance, the release of IL-6 in the LPWDPL-treated group gradually increased from 1009.42 pg/mL at the lowest concentration (125 μg/mL) and reached the maximum value of 1332.76 pg/mL at 500 μg/mL. In addition, LPWDPL significantly promoted NO generation. At concentrations of 125, 250, and 500 μg/mL, the NO generation of LPWDPL was 12.02 times, 10.79 times, and 22.44 times those of the control group, respectively (*p* < 0.05).

Taken together, LPWDPL, DDFVFLR, and LPVGPLFN peptides could significantly promote the secretion of pro-immune cytokines and the production of NO in RAW 264.7 macrophages, which demonstrates that they exhibit immunostimulatory activity, and the effects are concentration-dependent. The results also suggested that the three synthetic peptides may be used as immunomodulators. Despite this, its resistance to gastrointestinal digestion stability, effects in animals, and specific mechanisms of action still require further research.

## 4. Conclusions

In this study, *T. heterochaetus* was enzymatically hydrolyzed using flavorzyme to generate the hydrolysate product. Through ultrafiltration, liquid chromatography-mass spectrometry, and molecular docking techniques, three novel peptides, including LPWDPL, DDFVFLR, and LPVGPLFN, exhibiting immunomodulatory activities were identified in the hydrolysates. Analysis of amino acid composition indicated that the enzymatic hydrolysate was rich in amino acids closely related to biological activity, such as glutamic acid, aspartic acid, and lysine.

Among all ultrafiltration fractions, THPs-I exhibited the strongest immune-stimulating ability in vitro, significantly enhancing phagocytic activity of RAW 264.7 macrophages and promoting the secretion of TNF-*α*, IL-6, and NO. Notably, the immunostimulatory potency of THPs-I, particularly in inducing TNF-α and NO, appears comparable or superior to that reported for bioactive peptides from other sources, such as soybean protein hydrolysates and bullfrog (*Rana spinosa*) meat peptides, at similar concentrations. This enhanced activity may be attributed to the unique amino acid profile of *T. heterochaetus* peptides, especially their high content of hydrophobic and branched-chain amino acids, which are known to facilitate receptor interaction and membrane permeability, thereby potentially leading to more efficient macrophage activation. Molecular docking results showed that all three peptides could stably bind to the TLR4/MD-2 receptor through hydrogen bonds, hydrophobic interactions, and π-π stacking interactions. Cell experiments further confirmed that these peptides could effectively activate macrophages and promote the release of key immune mediators. Collectively, this study confirms the potential of *T. heterochaetus* as a source of immunomodulatory peptides. Their favorable safety profile (non-toxicity, non-allergenicity), predicted intestinal permeability, and potent in vitro immunomodulatory activity suggest two primary avenues for application: as functional ingredients in nutraceuticals or functional foods designed for immune support; and as lead compounds for the development of natural immune adjuvants or therapeutic agents aimed at modulating dysregulated immune responses.

While RAW 264.7 macrophages provide a valuable model for screening immunomodulatory activity, they cannot fully replicate the complexity of an intact immune system, including systemic metabolism, tissue distribution, and interplay between different immune cell types in vivo. In the future, the systemic immunomodulatory effects, optimal doses and safety of the identified peptides can be evaluated through animal studies (such as immunosuppressed mouse models). Or more detailed mechanism research can be conducted to confirm the participation of the signaling pathway proposed in the docking study. At the same time, in product development, formula strategies to enhance the stability, palatability and delivery of these peptides in potential functional foods or nutritional health products should be explored.

## Figures and Tables

**Table 1:** The optimal reaction conditions of enzymes.

Enzyme	Optimum Temperature (°C)	Optimum pH	Enzyme Dosage
alkaline protease	50	10.0	3%
neutral protease	50	7.0	3%
flavorzyme	50	7.0	3%
papain	55	7.0	3%
trypsin	37	7.5	3%
bromelain	55	7.5	3%

**Table 2:** Determination of Basic Nutritional Components of Fresh *T. heterochaetus*.

Samples	Protein (%)	Fat (%)	Ash (%)	Moisture Content (%)
*Tylorrhynchus heterochaetus*	12.54 ± 0.50	3.07 ± 0.05	0.69 ± 0.03	81.82 ± 0.05

**Table 3:** Amino acid composition of THPs.

Amino Acid	Content (g/100 g)
Asp	5.70
Thr *	2.68
Ser	1.82
Glu	8.19
Gly	3.82
Ala ^△^	4.29
Val ^△,^*	3.50
Met ^△^*	1.37
Ile ^△^*	3.18
Leu ^△^*	4.73
Tyr ^△^	1.65
Phe ^△^*	2.52
Lys *	5.13
His *	1.18
Arg	0.54
Pro ^△^	2.69
Total sum of hydrolyzed amino acids	52.90
Total sum of hydrophobic amino acids	24.70
Total sum of essential amino acids	24.29

Note: * indicates essential amino acids, and ^△^ indicates hydrophobic amino acids.

**Table 4:** Mw distribution of THPs.

M_W_ (Da)	Peak Area Ratio (%)	Retention Time (min)
180–500	21.80%	18.48
500–1000	57.65%	17.71
1000–3000	11.93%	16.99
3000–5000	8.13%	15.74
5000–10,000	0.28%	15.15

**Table 5:** Predicted physicochemical properties and binding energy of peptides with bioactivity scores ≥ 0.5.

Peptide	Binding Energy (kcal/mol)	Predicted Activity	ALC (%)	Toxicity	Allergenicity	Hydrophobicity	Caco-2 Cell Permeability
LPWDPL	−9.1	0.9351	98.7	None	None	0.10	−6.601
DDFVFLR	−9.0	0.8560	99.0	None	None	−0.13	−7.892
LPVGPLFN	−9.0	0.6596	99.5	None	None	0.20	−6.449
VPAPAP	−8.7	0.5485	98.1	None	None	0.14	−6.328
NPSRPW	−8.6	0.8573	98.2	None	None	−0.40	−6.399
LPGFP	−8.5	0.9595	98.5	None	None	0.23	−6.379
APWE	−8.5	0.7181	98.0	None	None	−0.02	−6.394
NAGDLFVHPR	−8.5	0.6514	98.0	None	None	−0.15	−7.519
HPNFNGN	−8.5	0.6353	99.4	None	None	−0.23	−6.046
YLGPK	−8.5	0.5502	98.9	None	None	−0.09	−6.615
LPPW	−8.4	0.9772	98.5	None	None	0.19	−5.882
LLPWVQ	−8.4	0.5009	99.1	None	None	0.2	−5.886
APFMDN	−8.3	0.8367	99.5	None	None	−0.05	−7.148
LDGLLEEHGPFL	−8.3	0.5724	99.4	None	None	0.05	−7.784
HPNFNGNTLDNDLMLLK	−8.3	0.5497	99.4	None	None	−0.15	−7.826
LPGGGP	−8.2	0.7618	99.2	None	None	0.15	−6.484
AHDDFLGK	−8.2	0.7291	98.4	None	None	−0.17	−7.71
VPFEDFGHAL	−8.2	0.6925	99.4	None	None	0.09	−7.487
AHPTFGK	−8.2	0.6247	98.5	None	None	−0.1	−6.837
LNPGGDRPLH	−8.2	0.5063	98.0	None	None	−0.23	−7.497
HPNF	−8.1	0.8757	98.2	None	None	−0.13	−6.148
KPDGPW	−8.1	0.8598	99.4	None	None	−0.24	−6.771
LPWQN	−8.1	0.7482	98.5	None	None	−0.1	−5.609
VAEGPNFLR	−8.1	0.6236	98.3	None	None	−0.11	−7.426
YPGQ	−8.1	0.5871	98.8	None	None	−0.14	−6.512
LPWGAN	−8.0	0.7084	99.3	None	None	0.1	−6.33
APGDYLLTLK	−7.9	0.7282	99.4	None	None	−0.01	−7.847
ALADFLR	−7.8	0.7848	98.5	None	None	−0.04	−7.658
TPPLLGDL	−7.8	0.5040	98.6	None	None	0.09	−7.554
WPGDLK	−7.7	0.8471	99.6	None	None	−0.14	−6.932
SDFLGLK	−7.7	0.7288	98.2	None	None	−0.04	−7.577
MPTFQ	−7.7	0.6502	98.6	None	None	−0.01	−6.617
LPWVQ	−7.7	0.5843	99.0	None	None	0.14	−5.957
TGWGK	−7.7	0.5399	98.7	None	None	−0.12	−6.797
WGSK	−7.6	0.6189	99.3	None	None	−0.21	−6.533
LPFSGM	−7.5	0.8552	99.4	None	None	0.20	−6.862
SPNGGGDPSGDLLELLK	−7.4	0.5837	99.8	None	None	−0.10	−9.167
YGPK	−7.3	0.5556	98.4	None	None	−0.25	−6.489
LPGGT	−7.3	0.5546	98.1	None	None	0.12	−6.764
AGFLEGGK	−7.2	0.5500	98.8	None	None	0.02	−7.464
LPGGP	−7.1	0.8168	99.1	None	None	0.14	−6.343
GPVGP	−7.1	0.6322	98.3	None	None	0.14	−6.65
MPAPLLE	−7.1	0.5655	98.5	None	None	0.12	−6.695

## Data Availability

The original contributions presented in this study are included in the article. Further inquiries can be directed to the corresponding authors.

## Abbreviations

*T. heterochaetus*, *Tylorrhynchus heterochaetus*; DMEM, Dulbecco’s modified eagle medium; PBS, Phosphate-buffered saline; FBS, Fetal bovine serum; CCK-8, Cell counting kit-8; DH, Degree of hydrolysis; THPs, Enzymatic hydrolysate of the *Tylorrhynchus heterochaetus*; THPs-I, Enzymatic hydrolysates of *Tylorrhynchus heterochaetus* with <3000 Da components; THPs-II, Enzymatic hydrolysates of *Tylorrhynchus heterochaetus* with >3000 Da components; Mw, Molecular weight; TFA, Trifluoroacetic acid; ACN, Acetonitrile; EP, Eppendorf; LC-MS/MS, Liquid chromatography-tandem mass spectrometry; HPLC, High-performance liquid chromatography; SD, mean ± standard deviation; BCAA, Branched-chain amino acid; NO, Nitric oxide; TNF-*α*, Tumor necrosis factor-alpha; IL-6, Interleukin-6; ALC, Acceptable level of confidence; TLR4/MD-2, Toll-like receptor 4/myeloid differentiation factor-2.

## References

[1] Zhao Y., Huang C., Xing J., Chen X., Zhang H. (2024). Ultrastructural observation of mature sperm and ovum of *Tylorrhynchus heterochaetus*. J. Fujian Agric. For. Univ. (Nat. Sci. Ed.).

[2] Yang Z., Sunil C., Jayachandran M., Zheng X., Cui K., Su Y., Xu B. (2019). Anti-fatigue effect of aqueous extract of Hechong (*Tylorrhynchus heterochaetus*) via AMPK linked pathway. Food Chem. Toxicol..

[3] Sunil C., Zheng X., Yang Z., Cui K., Su Y., Xu B. (2021). Antifatigue effects of Hechong (*Tylorrhynchus heterochaetus*) through modulation of Nrf2/ARE- mediated antioxidant signaling pathway. Food Chem. Toxicol..

[4] Chen M., Zhong X., Chen Y., Chen Y., Kong F., He Y. (2024). Optimization of enzymatic hydrolysis process for preparing polypeptide solution of *Tylorrhynchus heterochaetus* and investigation of its deodoration. J. Food Saf. Qual..

[5] Chen X., Mingming L., Heping L., Bo L., Liang G., Zining M., Lin H. (2016). Mitochondrial genome of the polychaete *Tylorrhynchus heterochaetus* (Phyllodocida, Nereididae). Mitochondrial DNA Part A.

[6] Gotoh T., Kamada S. (1980). Subunit Structure of Erythrocruorin from the Polychaete *Tylorrhynchus heterochaetus*. J. Biochem..

[7] Wu Y., Pang C., Wen S., Li H. (2006). Analysis and Evaluation of Nutritional Components in *Tylorrhynchus heterochaetus*. J. Water Conserv. Fish..

[8] Zeng D., Liu G., Dong J., Zeng X. (2025). Research Progress on the Nutritional Functions and Applications of *Tylorrhynchus heterochaetus*. Food Sci. Technol..

[9] Zeng Y., Cheng H., Zhong R., Zhong W., Zheng R., Miao J. (2025). Novel immunomodulatory peptides from hydrolysates of the Rana spinosa (*Quasipaa spinosa*) meat and their immunomodulatory activity mechanism. Food Chem..

[10] Zhou J., Chen M., Wu S., Liao X., Wang J., Wu Q., Zhuang M., Ding Y. (2020). A review on mushroom-derived bioactive peptides: Preparation and biological activities. Food Res. Int..

[11] Yu Y., Hu Q., Liu J., Su A., Xu H., Li X., Huang Q., Zhou J., Mariga A.M., Yang W. (2021). Isolation, purification and identification of immunologically active peptides from *Hericium erinaceus*. Food Chem. Toxicol..

[12] Yoo B.W. (2020). Embarking on a Career in Cardio-Rheumatology. J. Am. Coll. Cardiol..

[13] Zeng Y., Cheng H., Shen J., Lao L., Zheng R., Miao J. (2024). Response surface optimization of active peptides of Rana spinosa (*Quasipaa spinosa*) meat process and evaluation of immunomodulatory activity. J. Food Meas. Charact..

[14] Cai B., Chen H., Wan P., Luo L., Ye Z., Huang J., Chen D., Pan J. (2022). Isolation and identification of immunomodulatory peptides from the protein hydrolysate of tuna trimmings (*Thunnas albacares*). LWT.

[15] Khan A.I., Rehman A.U., Farooqui N.A., Siddiqui N.Z., Ayub Q., Ramzan M.N., Wang L., Xin Y. (2022). Effects of Shrimp Peptide Hydrolysate on Intestinal Microbiota Restoration and Immune Modulation in Cyclophosphamide-Treated Mice. Molecules.

[16] Shen C., Guo Z., Liang H., Zhang M. (2023). Preliminary investigation of the immune activity of PmH2A-derived antimicrobial peptides from the pearl oyster *Pinctada fucata martensii*. Fish Shellfish Immunol..

[17] He P., Zhang Y., Zhang Y., Zhang L., Lin Z., Sun C., Wu H., Zhang M. (2024). Isolation, identification of antioxidant peptides from earthworm proteins and analysis of the structure–activity relationship of the peptides based on quantum chemical calculations. Food Chem..

[18] Xia Z., Miao J., Chen B., Guo J., Ou Y., Liang X., Yin Y., Tong X., Cao Y. (2022). Purification, identification, and antioxidative mechanism of three novel selenium-enriched oyster antioxidant peptides. Food Res. Int..

[19] Zhang X., Zhang H., Jiao P., Xia M., Tang B. (2022). Preparation and Evaluation of Antioxidant Activities of Bioactive Peptides Obtained from Cornus officinalis. Molecules.

[20] Fu J.-j., He F.-y., Zhu X.-t., Wang Z., Huang Y.-n., Yu J.-x., Wu Z.-p., Chen Y.-w. (2024). Structural characteristics and antioxidant activity of Chinese giant salamander enzymatic hydrolysate-glucose conjugates induced by ultrasound Maillard reaction. Food Biosci..

[21] Zhao Z., Wang W., Chen J., Chen J., Deng J., Wu G., Zhou C., Jiang G., Guan J., Luo D. (2024). Effect of ultrasound-assisted Maillard reaction on functional properties and flavor characteristics of Oyster protein enzymatic hydrolysates. Ultrason. Sonochemistry.

[22] Jiang Y., Li S., Jiang L., Mu G., Jiang S. (2025). Immunomodulatory activity and molecular mechanisms of action of peptides derived from casein hydrolysate by alcalase and flavourzyme based on virtual screening. J. Dairy Sci..

[23] Wang Z., Liu Y., Zhang M., Tang Y., Yu M. (2024). Preparation and Characterization of Liposomes Encapsulating *Cyclina sinensis* Immunoreactive Peptides. J. Food Sci. Technol..

[24] Li Z., Wang S., Abou-Elsoud M., Li Y., Wang H., Liu M., Hu W., Ahn D.U., Huang X. (2025). Simulated Gastrointestinal Digestion Enhances the Immunomodulatory Activity of Ovalbumin Peptide NVMEERKIK: Mechanistic Insights into TLR4/MAPK/NF-κB Signaling Modulation. J. Agric. Food Chem..

[25] Lee C.H., Hamdan N., Nyakuma B.B., Wong S.L., Wong K.Y., Tan H., Jamaluddin H., Lee T.H. (2024). Purification, identification and molecular docking studies of antioxidant and anti-inflammatory peptides from Edible Bird’s Nest. Food Chem..

[26] Torres-Sánchez E., Martínez-Villaluenga C., Paterson S., Hernández-Ledesma B., Gutiérrez L.-F. (2025). Antidiabetic and Immunomodulatory Properties of Peptide Fractions from Sacha Inchi Oil Press-Cake. Foods.

[27] Wang L., Ma M., Yu Z., Du S.-k. (2021). Preparation and identification of antioxidant peptides from cottonseed proteins. Food Chem..

[28] Lee D.E., Shin G.R., Lee S., Jang E.S., Shin H.W., Moon B.S., Lee C.H. (2016). Metabolomics reveal that amino acids are the main contributors to antioxidant activity in wheat and rice *gochujangs* (Korean fermented red pepper paste). Food Res. Int..

[29] Neinast M., Murashige D., Arany Z. (2019). Branched Chain Amino Acids. Annu. Rev. Physiol..

[30] Gleeson M. (2005). Interrelationship between Physical Activity and Branched-Chain Amino Acids. J. Nutr..

[31] White P.J., Newgard C.B. (2019). Branched-chain amino acids in disease. Science.

[32] Chen M., Zhang F., Su Y., Chang C., Li J., Gu L., Yang Y. (2022). Immunomodulatory effects of egg white peptides on immunosuppressed mice and sequence identification of immunomodulatory peptides. Food Biosci..

[33] Kang H.K., Lee H.H., Seo C.H., Park Y. (2019). Antimicrobial and Immunomodulatory Properties and Applications of Marine-Derived Proteins and Peptides. Mar. Drugs.

[34] Li W., Ye S., Zhang Z., Tang J., Jin H., Huang F., Yang Z., Tang Y., Chen Y., Ding G. (2019). Purification and Characterization of a Novel Pentadecapeptide from Protein Hydrolysates of *Cyclina sinensis* and Its Immunomodulatory Effects on RAW 264.7 Cells. Mar. Drugs.

[35] Yang Q., Cai X., Huang M., Chen X., Tian Y., Chen G., Wang M., Wang S., Xiao J. (2020). Isolation, Identification, and Immunomodulatory Effect of a Peptide from *Pseudostellaria heterophylla* Protein Hydrolysate. J. Agric. Food Chem..

[36] Guo X., Liu J., Wang C., Wen Z., Zheng B. (2024). The Antioxidant Mechanism of Peptides Extracted from Tuna Protein Revealed Using a Molecular Docking Simulation. Antioxidants.

[37] Zhang Z., Shang Y., Li S., Chen Z., Xia J., Tian Y., Jia Y., Ma A. (2023). Molecular Docking Revealed the Potential Anti-Oxidative Stress Mechanism of the Walnut Polypeptide on HT22 Cells. Foods.

[38] Xu Y., Wang X., Liu Y., Du T., Yang H., Zhang H., Wang Y., Shen C. (2024). Optimization of Ultrasound-Assisted Enzymatic Hydrolysis of Immune Peptides from *Thunnus albacores*. Sci. Technol. Food Ind..

[39] He K., Zeng Y., Tian H., Zhang Z., Zhang H., Huang F., Yu F. (2021). Macrophage immunomodulatory effects of low molecular weight peptides from *Mytilus* coruscus via NF-κB/MAPK signaling pathways. J. Funct. Foods.

[40] Wen L., Jiang Y., Zhou X., Bi H., Yang B. (2021). Structure identification of soybean peptides and their immunomodulatory activity. Food Chem..

[41] Xu W., Zhao M., Fu X., Hou J., Wang Y., Shi F., Hu S. (2021). Molecular mechanisms underlying macrophage immunomodulatory activity of *Rubus chingii* Hu polysaccharides. Int. J. Biol. Macromol..

[42] Adler S.M., Paluska M.R., Svoboda K.R., Dallas D.C. (2024). Immunomodulatory bioactivities of glycomacropeptide. J. Funct. Foods.

[43] Kim H.-J., Kim H., Lee J.-H., Hwangbo C. (2023). Toll-like receptor 4 (TLR4): New insight immune and aging. Immun. Ageing.

[44] Ru Z., Xu M., Zhu G., Tu Y., Jiang Y., Du H. (2021). Ovotransferrin exerts bidirectional immunomodulatory activities via TLR4-mediated signal transduction pathways in RAW 264.7 cells. Food Sci. Nutr..

[45] Zhu L., Li W., Fan Z., Ye X., Lin R., Ban M., Ren L., Chen X., Zhang D. (2021). Immunomodulatory activity of polysaccharide from *Arca granosa* Linnaeus via TLR4/MyD88/NFκB and TLR4/TRIF signaling pathways. J. Funct. Foods.

[46] Han G., Xu Y., Li J., Li K., Xu X., Gao X., Zhao Y., Jiang H., Mao X. (2025). Hypoglycemic peptide preparation from *Bacillus subtilis* fermented with Pyropia: Identification, molecular docking, and in vivo confirmation. Food Chem..

[47] Fan H., Sun M., Li J., Zhang S., Tu G., Liu K., Xia Q., Jiang Y., Liu B. (2023). Structure characterization and immunomodulatory activity of a polysaccharide from Saposhnikoviae Radix. Int. J. Biol. Macromol..

